# Patent Foramen Ovale (PFO): History, Diagnosis, and Management

**DOI:** 10.31083/j.rcm2511422

**Published:** 2024-11-22

**Authors:** Aurel Maloku, Ali Hamadanchi, Albrecht Günther, Pawel Aftanski, P. Christian Schulze, Sven Möbius-Winkler

**Affiliations:** ^1^Department of Internal Medicine I, Cardiology, Angiology, Intensive Medical Care, University Hospital Jena, 07747 Jena, Germany; ^2^Department of Neurology, University Hospital Jena, 07747 Jena, Germany

**Keywords:** PFO, PFO-associated stroke, stroke, migraine

## Abstract

Current guidelines recommend closing a patent foramen ovale (PFO) in patients who have experienced a cryptogenic or cardioembolic stroke, have a high-risk PFO, and are aged between 16 and 60 years (class A recommendation, level I evidence). In terms of efficacy, in the CLOSE and RESPECT trials, the number needed-to-treat (NNT) to prevent one stroke recurrence in a five-year term was between 20 and 44. Other trials, such as the REDUCE trial, presented much better data with a NNT of 28 at two years and as low as 18 over a follow-up period of 10 years. Interventional PFO closure is relatively straightforward to learn compared to other cardiology procedures; however, it must be performed meticulously to minimize the risk of post-procedural complications. Usually, a double-disk occlusion device is used, followed by antiplatelet therapy. While the potential benefits of PFO closure for conditions such as migraines are currently being studied, robust trials are still required. Therefore, deciding to close a PFO for reasons other than stroke should be considered on a case-by-case basis.

## 1. Introduction 

A persistent foramen ovale (PFO) is found in 25% of the population [1, 2] and often presents without indication for interventional or surgical closure in most subjects. Therefore, careful patient selection is fundamental. There are several conditions where closure could be considered; the most important are in patients who suffered from a cryptogenic stroke and were found to have a PFO, the so-called PFO-associated stroke [3]. Evidence from recent large randomized prospective multicentric studies has shown a trend toward favoring PFO closure against medical therapy in patients with stroke and PFO [4, 5, 6]. In addition to PFO-associated stroke, there are several other conditions whereby, after careful patient selection, a PFO closure may be considered [7].

Our review summarizes the history of interventional PFO closure procedures and current guidelines.

## 2. History, Embryology, and Prevalence

Initially, PFO was described in one of the notebooks of the universal genius Leonardo da Vinci in 1513 [8]. During pregnancy, the patent foramen ovale (PFO) plays a crucial role as a shortcut for blood flow from the right to the left side of the heart. This bypass is necessary because the fetus’s lungs do not yet function for oxygen exchange. After birth, as the lungs begin to function, the need for this shortcut diminishes as intrapulmonary pressures and right ventricular afterload decrease, allowing transpulmonary blood to flow to the left atrium. Due to the change in intrathoracic pressure during the child’s first breaths, the PFO closes by increasing left atrial pressure. Typically, the two membranes of the atrial septum grow together within the first few years of life [9]; however, an open PFO is found in nearly a quarter of the general population.

In several studies, PFO occurrences in healthy subjects range from 27.3% in an autopsy study [1] to 25.6% in a transesophageal echocardiography study [2]. Patients with bicuspid aortic disease were found to possess PFOs up to 10 times more frequently [10]; however, gender [1] and race [11] do not influence prevalence.

## 3. PFO: Associated Conditions

Patent foramen ovale (PFO) is associated with various medical conditions. It has been linked to an increased risk of stroke, as blood clots bypass the venous system into the arterial system, thus entering the brain. Additionally, PFO is a concern for divers as it can lead to decompression sickness when bubbles form in the bloodstream during rapid ascents. Other related issues include migraine headaches and arterial deoxygenation syndromes, among several other potential health complications [12, 13, 14, 15, 16, 17, 18].

## 4. PFO and Stroke

A stroke is defined as an acute episode of focal dysfunction of the brain, retina, or spinal cord persisting for more than 24 hours or any duration if imaging or autopsy shows a corresponding infarction or hemorrhage [19]. However, despite careful investigation, the cause remains unknown in up to 25% of all strokes, described as cryptogenic strokes [20].

Stroke is the most debilitating of the PFO-associated conditions. Moreover, stroke is the second leading cause of death worldwide, closely behind ischemic heart disease [21]. The number of strokes and their complications increases annually [22]. A comparison of data from 1990 and 2010 showed a significant increase in stroke cases by up to 68%, with the majority being ischemic and hemorrhagic strokes [21, 22]. Additionally, the risk of recurrence of a stroke is quite high after the initial stroke, around 15% in the first two years [23].

## 5. PFO-Associated Stroke

The first ever described case of a PFO-related stroke was presented by Cohnheim in 1877. He performed an autopsy on a young patient and discovered a thrombotic occlusion of a cerebral artery. Further, the autopsy yielded a PFO; thus, he concluded that a blood clot had migrated from the venous system and via the PFO into the arterial circulation. That same thrombus had then been pumped to the cerebral artery and caused an occlusion. This is the first ever recorded case where a correlation was noted between a stroke and PFO [24]. After the report by Cohnheim, other cases of PFO-associated strokes followed, such as from Litten in 1880 [25]. Subsequent research and publications have demonstrated that a thrombus can migrate from the venous system and pass through PFO into the arterial system. This process illustrates how a PFO can be a conduit for potential complications between the two circulatory systems [26, 27].

Recently, six studies have been published addressing the management of a PFO-associated stroke, comparing medical therapy and interventional closure. The first three studies, which had an intermediate follow-up, published in 2012 and 2013 (CLOSURE-I, PC-Trial, RESPECT) showed no significant reduction in recurrent strokes in patients with interventionally closed PFO, compared to optimal medical therapy (OMT) [12, 13, 14]. In 2017 and 2018, three more studies were published (CLOSE, REDUCE, and DEFENSE), showing a significant reduction in stroke recurrence in the PFO closure group [4, 5, 6]. When the follow-up in the RESPECT study was prolonged to 6 years, it showed that PFO closure significantly reduced stroke recurrence compared to medical therapy [28]. The combined results of all six studies (follow-ups ranging from 24 to 71 months) showed a 75% reduction in recurrent stroke in patients under 60 years with a PFO (with a moderate-to-large and large right-to-left shunt) [29]. Moreover, several meta-analyses revealed that PFO closure reduces the risk of ischemic stroke, specifically in patients with cryptogenic stroke and a PFO [30, 31, 32].

## 6. PFO and Decompression Sickness

Decompression sickness (DCS) occurs due to a rapid change in pressure, either in altitude or rapid ascent from depth [15]. The condition is extremely rare, with a reported incidence rate of 2–5% [33]. The role of a PFO in decompression sickness remains debated; therefore, the decision to close the PFO must be made after a careful assessment of the individual. After a correlation between PFO and DCS was presented, the most important was lifestyle modification (body weight, smoking cessation, reduction of alcohol consumption, etc.). If the symptoms persist after the modification, then it should be suggested that the patient stop diving or flying; if this is not feasible, a PFO closure can be considered [15].

## 7. PFO and Migraine 

The role of PFO in migraines has long been postulated, and a recent meta-analysis showed a significant reduction in migraine attacks after PFO closure [16]. Furthermore, data showing a higher prevalence of PFO among migraine patients with aura [34] and the improvements in migraine attacks of patients who have undergone a PFO closure for other reasons [35] strengthen a possible correlation between PFO and migraine attacks. However, there is currently no recommendation for PFO closure in patients experiencing migraines. Moreover, routine PFO closure is advised against for migraine patients [15].

## 8. PFO and Arterial Deoxygenation Syndromes 

A narrowly selected group of patients with platypnea-orthodeoxia syndrome (POS) [17, 18] or obstructive sleep apnea syndrome (OSAS) [36] have shown potential for PFO closure. After closure, the arterial oxygen saturation increased, and the patient reported symptom relief. However, the consensus is that routine closure of the PFO should not be performed in these conditions [7].

## 9. PFO and Other Conditions

PFO may play a potential role in several conditions, such as stroke during pregnancy, perioperative stroke in non-cardiac surgery, and neurosurgery in the sitting position [7]; however, hard evidence remains limited.

## 10. Diagnosis of PFO 

**Fig. 1.**
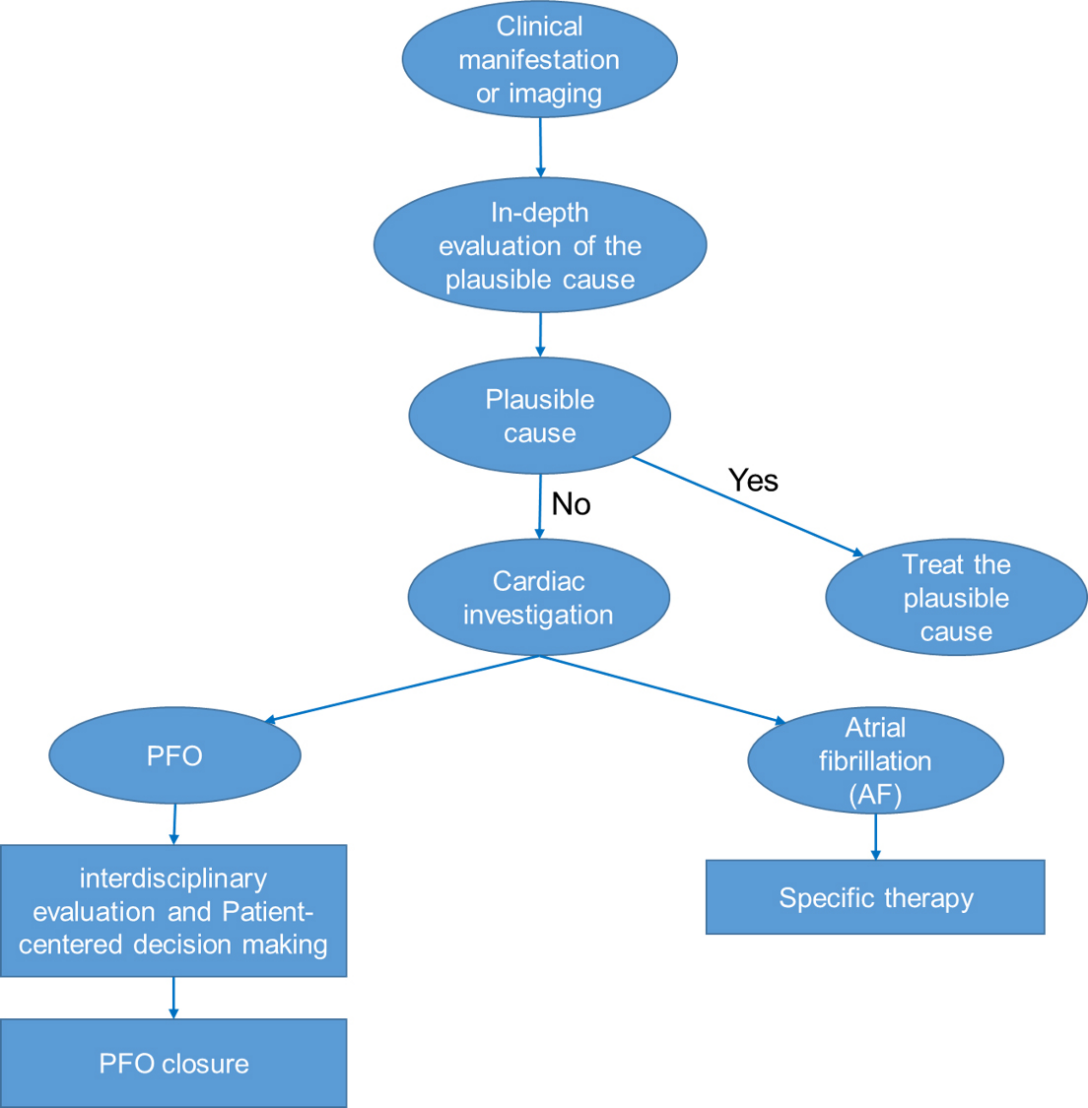
**Diagnostic chart, modified from Pristipino C *et al*. [3]**. PFO, patent foramen ovale.

The 2019 European position paper recommended that when a patient presents with a stroke or transient ischemic attack (TIA), a comprehensive investigation into the likely cause must be conducted. If the cause is neurovascular, it should be addressed as specified. If no clear results emerge from the neurovascular investigation, the focus should shift to a cardiac evaluation. This typically involves assessing two primary risk factors for intracardiac thrombi: Atrial fibrillation and patent foramen ovale (PFO) [3]. The management of intracardiac thrombi and atrial fibrillation is outlined in the relevant guidelines. The guidelines that guide this process include a diagnostic flow chart, which has been modified and is depicted in Fig. 1 (Ref. [3]).

Echocardiography is critical in diagnosis, risk stratification, and interventional planning for PFO closure [37, 38, 39, 40]. The most accurate method for determining the presence or absence of a PFO is a transesophageal echocardiogram (TEE), though lately, the role of a transthoracic echocardiogram (TTE) with an enhancing agent has come under focus [37]. The Stroke Update Conference in Stockholm recommended that, in patients with embolic strokes of undetermined etiology, a PFO screening should be performed using the bubble test-transcranial Doppler or TEE [41]. The above diagnostic methods have their advantages and disadvantages.

**Fig. 2.**
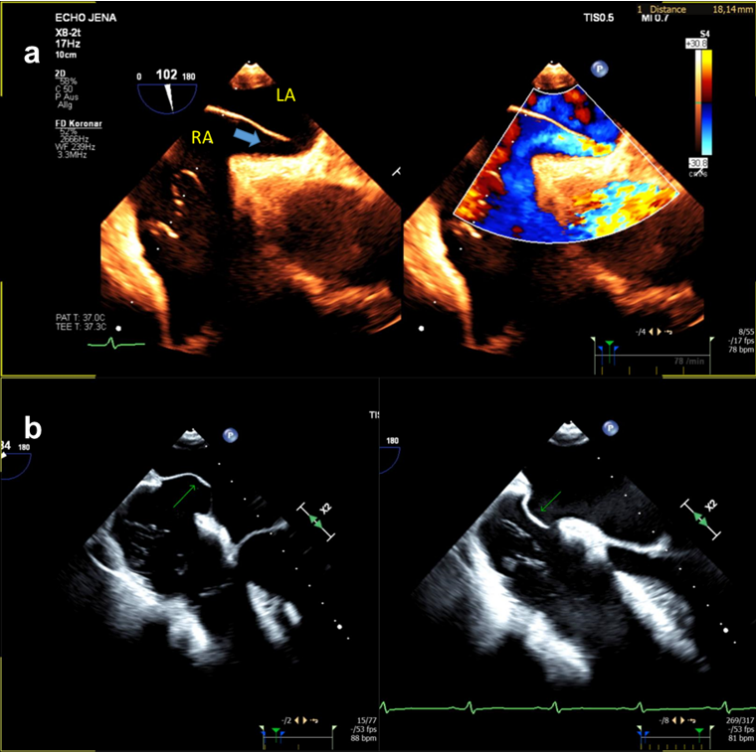
**Different echocardiographic manifestations of high risk PFO**. (a) long-tunnel PFO (blue arrow); (b) PFO with large atrial septal aneurysm (green arrow). RA, right atrium; LA, left atrium; PFO, patent foramen ovale; TEE, transesophageal echocardiogram.

A summary of the comparison between TTE and TEE, as well as their limitations, is summarized Table 1 (Ref. [42]), Table 2. On Fig. 2 are depicted two forms of high risk PFOs associated with increased stroke risk.

**Table 1.** **Comparison of TTE *vs.* TEE for the diagnostic evaluation of a PFO**.

Transthoracic *vs.* transesophageal echocardiography	
Transthoracic echocardiography	
Advantages	Disadvantages
∙ Well-tolerated, and no risk of complications	∙ Anatomical evaluation for procedural planning is not possible
∙ Relatively cheap and widely available	∙ Small shunts can be missed
∙ Has a high sensitivity (88%) and specificity (82%)	∙ Possibility of false positive due to intrapulmonary shunt
∙ Reasonable initial screening test	
Transesophageal echocardiography	
Advantages	Disadvantages
∙ Gold standard with a high sensitivity (89%) and high specificity (91%)	∙ More expensive
∙ A detailed anatomical evaluation of PFO can be performed	∙ Causes patient discomfort
∙ Vital for procedural planning	∙ Rare complications may occur

Modified from Filomena *et al*., 2021 [42]. PFO, patent foramen ovale; TTE, transthoracic echocardiogram; TEE, transesophageal echocardiogram.

**Table 2.** **Limitations of echocardiographic diagnoses and solutions based on our experience**.

Echocardiography limitations and solutions		
Pitfalls	Reasons	Solution
False negative bubbles test	- Inadequate Valsalva maneuver, promoting inadequate elevation of RA pressure	- Potential use of other contrast agents (e.g., Echovist®, Haemacell®, Gelifundol®)
	- Inadequate filling of RA with contrast material	- Elevate the forearm or apply abdominal pressure
	- Wash-out phenomenon by IVC flow	- Using the prominent IVC wash-out phenomenon, the contrast material should be injected at a lower extremity
Late appearance of contrast in LA	- Atypical PFO	- By applying gentle pressure on the patient’s abdomen, we can increase intra-abdominal pressure
	- Pulmonary AV fistula	
Atypical morphologies	- Multifenestrated septum with/without ASA	- Nyquist limit should be in the lower range (35–45 cm/sec) in color Doppler
	- Multiple small ASDs associated with PFO	- Three-dimensional imaging of the septum

IVC, inferior vena cava; ASDs, atrial septal defects; RA, right atrium; LA, left atrium; ASA, atrial septal aneurysm; AV, atrioventricular; PFO, patent foramen ovale.

Contrast-enhanced transcranial Doppler is a commonly used diagnostic tool; its main advantage is patient comfort. However, it requires a good cranial window [41].

Neurologists usually perform the patient referral after the patient has been treated for stroke or transient ischemic attack. The decision must be made by the Heart–Brain team.

## 11. Managing Conditions with a PFO

PFO closure represents a multifaceted challenge, evidenced by studies that both support and negate the benefits of the procedure. Given this challenging context and the pressing need for definitive guidelines to assist physicians in managing this condition, the European Association of Percutaneous Cardiovascular Interventions (EAPCIs) Scientific Documents and Initiatives Committee has spearheaded a collaborative effort of eight European scientific societies and international experts. The result was a position paper that addressed this problem [3].

The current German guidelines of three major societies *(Deutsche Gesellschaft für Neurologie, Deutsche Gesellschaft für Kardiologie, Deutsche Schlaganfall-Gesellschaft)* recommend the following:

- The RoPE-Score (including age, diabetes mellitus, hypertension, and hypercholesterolemia) may be used to estimate the role of PFO in cryptogenic stroke [43].
- Patients with prior cryptogenic ischemic attacks aged between 16 and 60 years should have the PFO closed [29] (level of recommendation A, class of evidence I, according to the Arbeitsgemeinschaft der Wissenschaftlichen Medizinischen Fachgesellschaften (AWMF) criteria [44]).
- The guidelines suggest using a disc-occluding device since it has been proven safer and more effective than non-circular disc-shaped occluding devices (level of recommendation A, class of evidence Ia) [29].
- Complications should not influence the decision to implant a PFO closure device (level of recommendation A, class of evidence Ia) [29].
- After the intervention, a dual antithrombotic therapy is recommended, with acetylsalicylic acid atrial septal aneurysm (ASA) (100 mg) and clopidogrel (75 mg) for 1–3 months. After the initial period, a monotherapy with ASA or clopidogrel for 12–24 months, when other conditions are present, lifelong monotherapy is recommended (level of recommendation B, class of evidence IIb) [29].
- To patients refusing a PFO closure, it should be made clear that there is no evidence which medical therapy is better, anticoagulation *vs.* antithrombotic treatment. Those patients should receive a secondary prevention strategy with ASA or clopidogrel (level of recommendation B, class of evidence IIb) [29].

## 12. PFO Occluders

The first ever reported case of successful interventional closure of a PFO dates back to 1987 [45]. Since then, several different devices, usually double umbrella occluders, have been developed. Initial experience with devices (ASDOS®, CardioSEAL®, StarFLEX®, PFO-Star®, Helex®, Angel Wings®, Amplatzer®) showed elevated risk for device-induced atrial fibrillation and elevated thrombogenicity [46]. However, the risks have been reduced significantly following the emergence of new generations [47].

## 13. Procedural Aspects of PFO Closure

Device implantation is typically performed in a standard cardiac catheterization laboratory, primarily utilizing fluoroscopy and TEE for guidance, along with physiological monitoring. There is some debate about the necessity of TEE, but it is widely used by interventionalists, particularly to reduce radiation exposure in younger patients. Combining both imaging techniques is recommended to maximize imaging clarity while minimizing radiation exposure. However, this approach requires consequent sedation (propofol or midazolam) to improve TEE tolerance. Further, patients need a peripheral line for fluid and medication administration, such as midazolam or propofol, continuous oxygen saturation, and arterial pressure monitoring.

The catheterization laboratory at the University Hospital Jena performs the procedure in accordance with expert recommendations and proceeds as follows:

1. The intervention is accessed through the femoral vein, typically on the right side. After the vein is punctured, a J-tip standard wire and a sheathless 5 Fr multipurpose (MP) catheter are inserted.
2. The right atrial pressure is measured to ensure it falls within the normal range of 6–12 mmHg. If it is lower, at least 500 mL of saline is administered.
3. The wire, aided occasionally by the MP catheter, is advanced through the PFO by pressing against the superior part of the atrial septum and usually passes through without difficulty.
4. In challenging cases, particularly with narrow or tunneled PFOs, TEE may assist in guiding the wire and catheter into the fossa ovalis. Optimal TEE visualization involves sweeping from 30° to 90° angles, with angiographic assistance typically provided in an anterior–posterior view, though a 45° left anterior oblique (LAO) view can be useful.
5. Once the left atrium is accessed, a 100 IU/kg body weight dose of heparin is administered, aiming for an activated clotting time of around 250 seconds. The left atrial pressure is then measured; if it is below 5 mmHg, intra venous (IV) fluids are administered to adjust the pressure to a safer range of 5–10 mmHg. The MP catheter is advanced under TEE guidance toward the left upper pulmonary vein, and a long, stiff guidewire with a soft tip is used to facilitate the exchange for a 24 mm sizing balloon. The necessity of balloon sizing is debated, but it helps determine the appropriate device size by assessing the septal opening, with measurements taken from two perpendicular echo projections and confirmed angiographically.
6. The selected closure device is prepared according to the manufacturer’s instructions, and a suitable sheath, typically between 7 and 9 Fr, is chosen based on the device size. After the sizing balloon is deflated and replaced with the sheath, a wet-to-wet connection ensures the bubble-free advancement of the device. The device is advanced into the sheath, then maneuvered into the mid-left atrium, where the left-sided disk is expanded and pulled back against the interatrial septum until slight resistance is felt. The sheath is then retracted over the device to deploy the right-sided disk, ensuring the device is firmly pressed against the septum.
7. Echocardiography confirms the device’s position, with the ‘packman sign’ observed angiographically indicating correct placement. The device should not compress the left atrial roof or the aorta. Once properly positioned, the device is released, and the delivery system and sheath are withdrawn. The femoral vein is closed using a Z-suture or a Proglide closure device.

Complications are rare but can include hematoma, atrioventricular (AV) shunt, stroke, TIA, pericardial effusion/tamponade, and device-associated atrial fibrillation [4, 5, 6, 12, 13, 14]. Pericardial tamponade from 0.2% to 0.4% [6, 12], cardiac perforation from 0.2% to 0.25% [4, 45], transient post-procedural atrial fibrillation/flutter from 0.5% to 5.4%, and device-associated thrombus occurred in 0% to 1.1%, while bleeding as complication ranged from 0% to 2.5% [4, 5, 6, 12, 13, 43].

## 14. Conclusions

PFOs should be closed in patients suffering from a cryptogenic stroke, aged between 16 and 60 years, to prevent stroke recurrence.
